# Westwards and northwards dispersal of *Triosteum himalayanum* (Caprifoliaceae) from the Hengduan Mountains region based on chloroplast DNA phylogeography

**DOI:** 10.7717/peerj.4748

**Published:** 2018-05-11

**Authors:** Hai-rui Liu, Qing-bo Gao, Fa-qi Zhang, Gulzar Khan, Shi-long Chen

**Affiliations:** 1 Key Laboratory of Adaptation and Evolution of Plateau Biota, Northwest Institute of Plateau Biology, Chinese Academy of Sciences, Xining, China; 2 Qinghai Provincial Key Laboratory of Crop Molecular Breeding, Xining, China; 3 University of Chinese Academy of Sciences, Beijing, China

**Keywords:** Chloroplast DNA, Palaeodistributional reconstruction, Himalaya–Hengduan Mountains, Phylogeography, Range expansion

## Abstract

The varying topography and environment that resulted from paleoorogeny and climate fluctuations of the Himalaya–Hengduan Mountains (HHM) areas had a considerable impact on the evolution of biota during the Quaternary. To understand the phylogeographic pattern and historical dynamics of *Triosteum himalayanum* (Caprifoliaceae), we sequenced three chloroplast DNA fragments (*rbcL-accD*, *rps15-ycf1*, and *trnH-psbA*) from 238 individuals representing 20 populations. Nineteen haplotypes (H1–H19) were identified based on 23 single-site mutations and eight indels. Most haplotypes were restricted to a single population or neighboring populations. Analysis of molecular variance revealed that variations among populations were much higher than that within populations for the overall gene pool, as well as for the East Himalayan group (EH group) and the North Hengduan group (NHM group), but not for the Hengduan Mountains group (HM group). Ecoregions representing relatively high genetic diversity or high frequencies of private haplotypes were discovered, suggesting that this alpine herbaceous plant underwent enhanced allopatric divergence in isolated and fragmented locations during the Quaternary glaciations. The current phylogeographic structure of *T. himalayanum* might be due to heterogeneous habitats and Quaternary climatic oscillations. Based on the phylogeographic structure of *T. himalayanum* populations, the phylogenetic relationship of identified haplotypes and palaeodistributional reconstruction, we postulated both westwards and northwards expansion from the HM group for this species. The westwards dispersal corridor could be long, narrow mountain areas and/or the Yarlung Zangbo Valley, while the northwards movement path could be south–north oriented mountains and low-elevation valleys.

## Introduction

The present population genetic structure of species carries signals of past dynamics (Hewitt, 2000). Exploration of the interaction between environmental heterogenization, climate changes, and biota evolution can help us understand how organisms have been influenced by various environmental and climatic events. The Himalayas and Hengduan Mountains are both biodiversity hotspots of the Northern Hemisphere. The Himalaya–Hengduan Mountains (HHM) region, which extends along the southern frontier to the southeastern rim of the Qinghai–Tibetan Plateau (QTP), contains more than 20,000 species of vascular plants and harbors most plant families and genera of Eurasian flora with a high frequency of endemics (Wu, 1988; Li & Li, 1993). The HHM is also considered to be the core area of the Sino-Himalayan flora (Wu & Wu, 1996) as well as the distribution and diversification center for many alpine plants (Sun, 2002). Moreover, this region has been regarded as the origin center of boreal-temperate plants (Li & Li, 1993; Gao et al., 2012a; Jia et al., 2012; Lu et al., 2014; Favre et al., 2015).

In the early Eocene, the Indian Plate collided with the Eurasian Plate, directly resulting in subsequent uplift of the QTP and the Himalayas and Hengduan Mountains (Zheng & Yao, 2004; Chatterjee, Goswami & Scotese, 2013). The significant orogeny of Himalayas likely occurred during the Miocene (Wang et al., 2008), although the onset of Himalayan orogeny may have started slightly earlier (Chatterjee, Goswami & Scotese, 2013; Gébelin et al., 2013). The elevation history of the QTP suggests that the orogeny of the Hengduan Mountains occurred as a final phase of the uplift after 10 Ma (Mulch & Chamberlain, 2006). Sun et al. (2011) concludes that the Hengduan Mountains underwent a major uplift only after the Miocene, attaining its peak elevation shortly before the Late Pliocene according to paleobotanical and paleoclimatic data. Consequently, long, narrow east–west oriented mountainous areas and valleys (e.g., the Yarlung Zangbo Valley) of the Himalayan Mountains were formed, while north–south oriented valleys surrounded by high peaks of the Hengduan Mountains appeared.

Although geological records show that no unified ice sheets were developed in QTP and adjacent regions during the Quaternary period (Li & Pan, 2002), arid-cold glacial and wet-warm interglacial periods greatly affected the distribution range and diversification of plant species in this region. In fact, regional expansions and intraspecific divergences during the Quaternary climatic oscillations are very common in most studied alpine and subalpine species of the HHM (Liu et al., 2012; Yu et al., 2017, and references therein).

Thus, the unique, abundant, and diverse flora of the HHM, resulting from topographical diversity and climate fluctuation, have made this area one of the most prevalent regions for phylogeographic survey. Numerous hypotheses have been provided based on different phylogeographic patterns. However, previous studies mainly focused on the tree (Opgenoorth et al., 2010; Li et al., 2013; Liu et al., 2013; Sun et al., 2014) and shrub species (Wang et al., 2009; 2010; Jian, Tang & Sun, 2015; Gao et al., 2015), while only a few studies have focused on herbaceous plant species (Zhang, Volis & Sun, 2010; Gao et al., 2012b), especially those that grow in the humid habitats of the HHM region.

*Triosteum himalayanum* Wallich (Caprifoliaceae) is a perennial herb with a distribution in the eastern Himalayas, Hengduan Mountains, and central China (including Tibet, Yunnan, Sichuan, Hubei, Shaanxi, and Henan) at elevations between 1,800 and 4,100 m a.s.l. This species shows a fragmented distribution, occupying relatively wet habitats on mountain slopes, coniferous forests, streamsides, and grasslands (Yang et al., 2011). Gould & Donoghue (2000) conducted a phylogenetic and biogeographic study on the genus *Triosteum* L. based on morphological features and sequence polymorphism of the nuclear DNA internal transcribed spacer and granule-bound starch synthase gene (GBSSI or *waxy*), which provided the biogeographic pattern of *Triosteum* across North America and eastern Asia. However, they presented no details of the intraspecific variation and biogeography of *T. himalayanum* in the HHM and adjacent regions. A phylogeographic study of *T. himalayanum* would give us a better understanding of how fragmented populations have evolved since species diversification in the context of environmental heterogenization and Quaternary glaciation. Furthermore, a phylogeographic survey of multiple co-distributed species (e.g., the coniferous trees *Taxus wallichiana*, *Picea likiangensis*, and *Juniperus tibetica*) would be greatly useful for providing a detailed picture of plant diversity and endemism of regional hotspots (Qiu, Fu & Comes, 2011). Here, we employed three chloroplast DNA (cpDNA) sequences, *rbcL-accD*, *rps15-ycf1*, and *trnH-psbA*, combined with palaeodistributional reconstruction modeling analysis to infer phylogeography and demography dynamics of *T. himalayanum*. The detailed aims of this study are: (1) to reveal the genetic structure of this species with fragmented distribution and (2) to infer the historical dynamics, especially the dispersal direction between ecogroups and pathways in association with habitat differentiation and Quaternary climate fluctuations. In order to better interpret these issues, we divided all the sampled populations into three groups: the East Himalayan group (EH group, including populations P1–P7), the Hengduan Mountains group (HM group, P8–P12), and the north Hengduan Mountains group (NHM group, including P13–P20).

## Materials and Methods

### Material sampling

**Figure 1:**
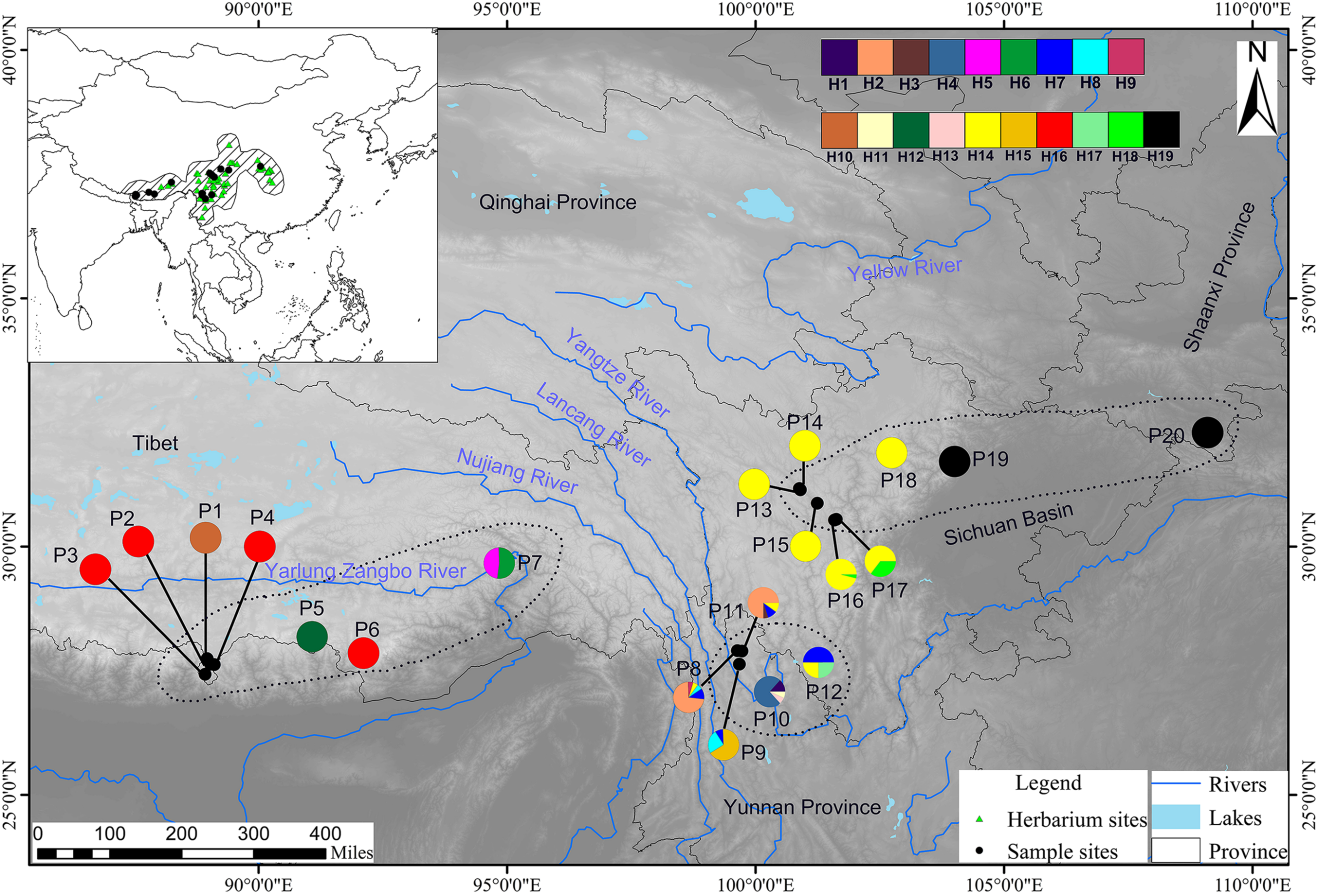
Geographic distribution of cpDNA haplotypes (H1–H19) detected among 20 populations (for population codes see Table 1) and herbarium sites of *T. himalayanum*. Pie charts show the proportion of haplotypes within each population.

Field expeditions were conducted between 2006 and 2015 to cover all of the possible distribution localities of *T. himalayanum* according to specimen records in the herbaria. However, populations of *T. himalayanum* that were previously distributed in central China (according to the specimen study) were difficult to collect due to land use changes. Fresh leaves of 4–25 individuals were collected for each population, separated at least 20 m apart. A total of 238 individuals from 20 populations were collected. Among these populations, 19 were from the HHM, and only one population (P20) was not from this region (Table 1; Fig. 1). Leaf materials were dried in silica gel and stored at room temperature. Voucher specimens of all populations are deposited in the Herbarium of Northwest Institute of Plateau Biology (HNWP), Xining, Qinghai, China.

**Table 1:** Details of sample locations and sample sizes of 20 *T. himalayanum* populations.

POP	Locality	Voucher no.	Latitude (N)	Longitude (E)	Altitude (m)	Sample size	Haplotypes (no. of individuals)
P1	Yadong, T	Chen2013450	27°45′	88°58′	3,065	6	H10(6)
P2	Yadong, T	Chen2014519	27°38′	89°02′	4,200	5	H16(5)
P3	Yadong, T	Chen2014520	27°26′	88°55′	2,915	16	H16(16)
P4	Yadong, T	Chen2013464	27°37′	89°07′	4,771	12	H16(12)
P5	Luozha, T	Chen2013390	28°05′	91°05′	4,249	8	H12(8)
P6	Cuona, T	Chen2014443	27°48′	91°59′	3,720	4	H16(4)
P7	Luozha, T	Chen2014305	29°42′	94°44′	3,500	19	H5(9) H6(10)
P8	Xianggelila, YN	Chen2012111	27°54′	99°38′	3,380	18	H2(13) H7(2) H8(1) H9(1) H14(1)
P9	Xianggelila, YN	Chensl1849	27°38′	99°40′	3,580	12	H7(1) H8(3) H15(8)
P10	Yulong, YN	Chensl1846	27°03′	100°12′	3,420	15	H1(2) H4(11) H11(1) H13(1)
P11	Xianggelila, YN	Chen2013578	27°54′	99°44′	3,331	20	H2(15) H3(1) H7(2) H14(2)
P12	Yanyuan, SC	Chen2013519	27°41′	101°13′	3,240	4	H7(2) H14(1) H17(1)
P13	Luhuo, SC	Chen2013129	31°10′	100°53′	3,080	7	H14(7)
P14	Luhuo, SC	Chen06310	31°09′	100°54′	3,060	10	H14(10)
P15	Daofu, SC	Chensl1879	30°52′	101°15′	3,380	16	H14(16)
P16	Danba, SC	Chensl1881	30°32′	101°35′	3,810	25	H14(24) H18(1)
P17	Danba, SC	Chen06127	30°33′	101°38′	3,410	17	H14(11) H18(6)
P18	Hongyuan, SC	Chensl1840	31°50′	102°41′	3,370	12	H14(12)
P19	Maoxian, SC	Chen06084	31°40′	103°56′	3,500	4	H19(4)
P20	Langao, SX	Zhang2015014	32°17′	109°04′	2,130	8	H19(8)

**Notes:**

For each population, haplotype composition (H1–H19) is indicated.

YN, Yunnan; SC, Sichuan; SX, Shaanxi; T, Tibet.

*Lonicera trichosantha* Bureau & Franchet (Caprifoliaceae), has been chosen as out group for haplotype phylogenetic analyses.

### DNA extraction, polymerase chain reaction amplification, and sequencing

Total DNA was extracted from the silica-gel dried leaf materials following a modified CTAB (Cetyltrimethyl Ammonium Bromide; Doyle & Doyle, 1987). Samples were genetically screened using the universal cpDNA primers described by Hamilton (1999) and Dong et al. (2012). After preliminary screening (pilot screening) of more than 10 intergenic spacers, *rbcL-accD*, *rps15-ycf1*, and *trnH-psbA* were chosen for further analysis, since these three intergenic spacers revealed sequence polymorphism. Polymerase chain reactions (PCRs) were performed in 25 μL volume containing 0.6 μL of template DNA (approximately 20 ng), 2.5 μL of 10 × PCR buffer (with Mg^2+^), 1.0 μL of 10 mM dNTPs, 0.5 μL of 5 pM of each primer, and 0.2 μL (1.5 units) of Taq polymerase (Takara, Dalian, China). Amplification conditions were: 5 min at 95 °C; followed by 35 cycles of 50 s at 95 °C, 1 min at 58 °C, and 1 min at 72 °C; with a final extension of 6 min at 72 °C. PCR products were purified using a CASpure PCR Purification Kit (CASarry, Shanghai, China) following the manufacturer’s protocols. Sequencing reactions and analysis were performed using an ABI 3730xl DNA sequencer (Applied Biosystems, Foster City, CA, USA). Primers used for sequencing were the same as those used for amplification.

### Population genetic and phylogeographic analysis

The chromatograms of each sequence were checked visually using Chromas ver. 2.33 (http://www.technelysium.com.au). DNA sequences were aligned in Clustal_X (Thompson et al., 1997) and then checked by visual inspection with minor adjustment. The cpDNA haplotypes were identified by DnaSP ver. 5.0 (Librado & Rozas, 2009). All newly generated sequences from *T. himalayanum* were submitted to GenBank, and the access numbers are MH122760–MH122783. Three cpDNA fragments, *rbcL*-*accD*, *rps15*-*ycf1*, and *trnH*-*psbA*, were then concatenated for the subsequent analyses. In the following analysis, we did not code the indels; each indel was considered as a one-step mutation.

The spatial genetic structure of cpDNA haplotypes was analyzed to define groups of populations that were geographically homogeneous and maximally differentiated from each other using the program SAMOVA ver. 1.0 (Dupanloup, Schneider & Excoffier, 2002); the number of initial conditions was set to 100, and pairwise difference (DNA) was chosen as the molecular distance.

Estimates of average gene diversity within populations (*H*_S_), total gene diversity (*H*_T_), *G*_ST_ (Nei, 1987), and *N*_ST_ (Grivet & Petit, 2002) were calculated for the overall populations and for each regional group using PERMUT ver. 2.0 (Pons & Petit, 1996). *G*_ST_ and *N*_ST_ were used to estimate population differentiations; *G*_ST_ only takes haplotype frequency into account, whereas *N*_ST_ also takes sequence similarities between haplotypes into account. A permutation test with 1,000 permutations was used to compare *G*_ST_ and *N*_ST_. Significantly higher values of *N*_ST_ than the estimated *G*_ST_ indicated the presence of a phylogeographical structure (Pons & Petit, 1996).

Analysis of molecular variance (AMOVA; Excoffier, Smouse & Quattro, 1992) for overall populations and each regional group of *T. himalayanum* was conducted using ARLEQUIN version 3.11 (Excoffier, Laval & Schneider, 2005). The *F*-statistic (*F*_ST_) was calculated, and significance was tested.

### Phylogenetic analyses

The appropriate model of DNA substitution for maximum likelihood (ML) analysis was determined using the AIC in jModelTest ver. 2.1.6 (Darriba et al., 2012; Guindon & Gascuel, 2003). Best-fit models for the cpDNA datasets were determined to be HKY+G. ML trees of cpDNA haplotypes of *T. himalayanum* were generated in PhyML ver. 3.1 (Guindon & Gascuel, 2003) using *L. trichosantha* as the outgroup. We selected BioNJ as the distance-based tree reconstruction method during tree searching. Subtree pruning and regrafting (SPR) moves were relied on to explore the space of tree topologies. The number of random starting trees was five. The tree branch support was estimated by non-parametric bootstrap analysis, and the number of bootstrap replicates was 100. Bayesian inference was conducted using MrBayes ver. 3.2 (Ronquist et al., 2012). The MCMC algorithm was run for 10,000,000 generations starting from random trees, and one in every 100 generations was sampled. The first 25% of the trees was discarded as burn-in followed by reconstruction of the majority consensus tree. Internodes with posterior probabilities >95% were considered to be statistically significant. When the average standard deviation of split frequencies between the two independent runs was smaller than 0.01, the analysis was stopped. Maximum parsimony (MP) analysis was implemented using Phylip ver. 3.69 (Felsenstein, 1985, 1988; Margush & McMorris, 1981). The number of bootstrap replicates was set to 1,000, and the random number seed was five.

To further detect genealogical relationships, a haplotype network was constructed among cpDNA haplotypes using the median‐joining method and MP calculations as implemented in Network ver. 5.0.0.0 (Bandelt, Forster & Röhl, 1999; Polzin & Daneschmand, 2003; http://www.fluxus-engineering.com).

### Demographic analyses

For the overall populations and three regional groups, Tajima’s *D* and Fu’s *Fs* of neutrality tests with 1,000 simulated samples were performed using ARLEQUIN ver. 3.11 (Fu, 1997). Negative values are expected when there have been recent population expansions or population bottlenecks (Tajima, 1989). Pairwise mismatch distribution analysis (Rogers & Harpending, 1992) for overall populations and each group based on the cpDNA dataset was performed assuming historical population dynamics using DnaSP ver. 5.0 (Librado & Rozas, 2009). Unimodal distributions reflect rapid growth from small populations, while multimodal distributions reflect long-term population stability (Slatkin & Hudson, 1991; Rogers & Harpending, 1992).

### Palaeodistributional reconstruction

Potential geographic ranges and the effects of past climatic oscillations for *T. himalayanum* has computed across current, Mid Holocene (6 ka), last glacial maximum (LGM; 22 ka), and last interglacial maximum (LIG; 130 ka, Otto-Bliesner et al., 2006). We simulated all the species distribution models, i.e., GBM (generalized boosted models; Ridgeway, 2006), SRE (surface range envelop; Busby, 1991), GLM (generalized linear models; Peter & John, 1989), CTA (classification tree analysis; Breiman, 2001, 2017), ANN (artificial neural network; Ripley, 1996), FDA (flexible discriminant analysis; Hastie, Tibshirani & Buja, 1994), MARS (multivariate adaptive regression splines; Friedman, 1991), RF (random forests; Breiman, 2001), and MAXENT (maximum entropy; Phillips, Dudík & Schapire, 2004) in R packages “Biomod 2” (Thuiller, Georges & Engler, 2014), supported with rworld map, rgdal, dismo, SDMTools. The effectiveness of all the models were evaluated using TSS (true skill statistics) and AUC (area under curve; curve-receiver operating characteristic curve) values >0.7. A total of nine bioclimatic variables (BIO4 = temperature seasonality, BIO5 = max temperature of warmest month, BIO6 = min temperature of coldest month, BIO7 = temperature annual range, BIO8 = mean temperature of wettest quarter, BIO9 = mean temperature of driest quarter, BIO10 = mean temperature of warmest quarter, BIO11 = mean temperature of coldest quarter, and BIO15 = precipitation seasonality) showed low correlation and high informativeness after a jackknife procedure on the 19 BIOCLIM variables downloaded from the WorldClim data set (Hijmans et al., 2005).

## Results

### Haplotype variation and distribution

The combined cpDNA sequences of *rbcL-accD*, *rps15-ycf1*, and *trnH-psbA* of *T. himalayanum* ranged from 1,698 to 1,718 bp in length, with an alignment length of 1,727 bp. Nineteen haplotypes (H1–H19) were identified based on polymorphic sites across the concatenated cpDNA sequence data (Table 2). Among those polymorphic sites, we found 23 single-site mutations and eight indels. The *rbcL-accD* region was the most variable fragment (14 substitutions; three indels), followed by *rps15–ycf1* (six substitutions; five indels), and *trnH-psbA* (three substitutions).

**Table 2:** Sequence polymorphisms detected in three chloroplast DNA regions of *T. himalayanum* identifying 19 haplotypes (H1–H19).

Nucleotide position	*rbcL-accD*																	*rps15-ycf1*											*trnH*-*psbA*		
			2	3	4	4	5	5	6	6	6	6	6	6	6	6	6				2	2	3	3	4	4	4	4	1	1	2
	1	5	0	3	5	6	4	6	1	1	1	2	2	2	2	2	5	2	7	9	4	9	0	8	2	4	4	4	2	7	1
	3	2	5	9	0	7	9	9	3	4	5	1	2	4	5	6	5	4	9	8	6	8	9	4	3	5	8	9	8	7	1
H1	A	G	G	C	G	C	–	G	T	A	A	A	A	–	T	T	*	C	d	–	A	A	T	T	–	G	–	–	G	G	C
H2	·	·	·	·	·	·	–	·	·	·	·	·	·	$	·	·	·	A	·	–	·	·	·	·	–	·	–	–	·	·	·
H3	·	·	·	·	·	·	–	·	·	·	·	·	·	$	·	·	·	A	·	–	·	·	·	·	–	·	–	–	·	T	·
H4	·	·	·	·	·	·	–	·	·	·	·	·	·	–	·	·	·	A	·	–	·	·	·	·	–	·	–	–	·	·	·
H5	T	·	A	·	A	·	–	·	·	·	·	·	·	$	A	·	–	A	·	–	·	C	·	G	–	·	ψ	–	T	·	·
H6	T	·	·	·	A	·	–	·	·	·	·	·	·	$	A	·	–	A	·	–	·	C	·	G	–	·	ψ	–	T	·	·
H7	·	·	·	·	·	·	–	·	·	·	·	·	·	$	A	·	·	A	·	–	·	·	·	·	–	·	–	–	·	·	·
H8	·	·	·	·	·	·	–	·	·	·	·	·	·	$	A	A	·	A	·	–	·	·	·	·	–	·	–	–	·	·	·
H9	·	A	·	·	·	·	–	·	·	·	·	·	·	$	A	·	·	A	·	–	·	·	·	·	–	·	–	–	·	·	·
H10	·	·	·	·	·	·	–	·	A	·	·	·	·	$	A	·	·	A	§	–	·	·	·	·	–	·	–	–	·	·	·
H11	·	·	·	·	·	·	#	·	A	·	·	T	·	$	A	·	·	A	·	–	·	·	·	·	–	·	–	–	·	·	·
H12	·	·	·	·	·	·	–	·	A	·	·	·	T	$	A	·	·	A	·	–	·	·	·	·	–	·	–	–	·	·	·
H13	·	·	·	·	·	·	–	·	·	·	·	·	–	$	A	·	·	A	·	–	·	·	·	·	–	·	–	–	·	·	·
H14	·	·	·	·	·	·	–	·	·	·	·	·	T	$	A	·	·	A	·	–	·	·	·	·	–	·	–	–	·	·	·
H15	·	·	·	·	·	·	–	·	·	·	·	·	T	$	A	·	·	A	·	–	·	·	·	·	–	·	–	–	·	T	·
H16	·	·	·	·	·	T	–	·	·	·	·	·	T	$	A	·	·	A	·	–	·	·	·	·	–	·	–	–	·	·	·
H17	·	·	·	·	·	·	–	·	·	·	·	·	T	$	A	·	·	A	·	–	·	·	·	·	ξ	·	–	–	·	·	·
H18	·	·	·	·	·	·	–	·	·	·	·	·	T	$	A	·	·	A	–	–	·	·	·	·	–	·	·	–	·	·	·
H19	·	·	·	A	·	·	#	A	T	T	T	·	·	$	A	·	·	A	–	ж	G	·	G	·	–	A	ψ	&	T	·	A

**Notes:**

Sequences are numbered from the 5′ to the 3′ end in each region.

# = AGGTCATGTGCCAC, $ = A, * = TCTC, § = CGAATT, ж = T, ξ = ATATC, ψ = G, & = GG.

Twelve haplotypes (accounting for 63.2% of the total haplotypes) were relatively rare, each was restricted to a single population (H1, H3–H6, H9–H13, H15, and H17). Moreover, all of the twelve haplotypes were distributed in the HM group of five populations, indicating a very high level of molecular diversity (Table 1). Another seven haplotypes were shared by at least two populations, suggesting a substantial amount of population isolation at the cpDNA level.

The most common haplotype was H14, which occurred in nine populations with variable locations in the HM group (three populations) and NHM group (six populations). Haplotypes H7 and H16 both occurred in four populations but with locations not far from each other (Fig. 1).

### Population variation and phylogeographic structure

Twelve populations (P1–P6, P13–P15, and P18–P20) were fixed for single haplotype, showing no variation within the population. However, the remaining eight populations (P7–P12, P16, and P17) in the HM and NHM groups were particularly diverse, with two to five haplotypes in each population (Table 1; Fig. 1). Populations P1–P6 were found to belong to the EH group; each contained only one haplotype, and all three haplotypes present in these six populations were endemic.

Population P19 was located far from P20, isolated by the Sichuan Basin. However, these two populations shared the sole haplotype H19.

SAMOVA failed to detect any meaningful geographical groups based on cpDNA haplotypes. *K* values increased from two to seven while *F*_CT_ values changed irregularly from 0.897 to 0.781. However, each newly defined group was represented by a single population. On the basis of the distribution ecoregions of the populations and the genetic structure of the *T. himalayanum* populations, we divided the populations into three groups: the East Himalayan group (EH group, including populations P1–P7), the Hengduan Mountains group (HM group, P8–P12), and the North Hengduan Mountains group (NHM group, including P13–P20). The division backgrounds which can be considered as temporal and biota differences between these three eco-regions are illustrated as follows: (1) distinctions between the Hengduan Mountains and the Himalaya. Mulch & Chamberlain (2006) suggests that the orogeny of the Hengduan Mountains occurred as a final propagation of the palaeoelevation history of the QTP uplift after 10 Ma, reflecting its much younger age than the Himalaya. In addition, the HM is more species-rich compared with the Himalaya, and biota diversification process of HM is distinguishing from that of Himalaya (Xing & Ree, 2017); (2) differences between the NHM group and HM group-even the NHM group occupies part of the Hengduan Mountains and adjoining highland, the genetic diversity of *T. himalayanum* and some other species (Du et al., 2017) in there is quite lower than that of core area of Hengduan Mountains (HM group). A similar grouping strategy can be found in many studies of plant species that are sympatric with *T. himalayanum* (Sun & Zhou, 1996; Zou et al., 2012; Liu et al., 2013; Yu et al., 2015; Gao et al., 2016; Du et al., 2017). The EH group was distributed along the Yarlung Zangbo River or Himalayan Mountains, ranging from the big bend gorge of Yalutsangpu to the Yadong valley. The HM group is located in the core region of the Hengduan Mountains with rich molecular diversity. The location of the NHM group is the northern part of the Hengduan Mountains region, which is also the eastern edge of the QTP.

For the overall populations, the *N*_ST_ value was 0.929, while the *G*_ST_ value was 0.781, and the difference between them was significant (*p* < 0.001). A significant difference between *N*_ST_ and *G*_ST_ was also found in the EH and NHM groups but not in the HM group. The values of total genetic diversity (*H*_T_) were much higher than those of gene diversity within populations (*H*_S_) for *T. himalayanum* in the overall populations (*H*_T_ = 0.878, *H*_S_ = 0.192), EH group (*H*_T_ = 0.714, *H*_S_ = 0.075), HM group (*H*_T_ = 0.922, *H*_S_ = 0.550), and NHM group (*H*_T_ = 0.498, *H*_S_ = 0.071, Table 3). AMOVA for the overall populations revealed that 89.43% of the total genetic variation occurred among populations, compared with 10.57% within populations; the pairwise *F*_ST_ value was 0.8943. Similar AMOVA results were obtained for both the EH group and NHM group. However, the HM group exhibited a higher level of within-population variation compared with among-population variation (Table 4).

**Table 3:** Estimates of average gene diversity within populations (*H*_S_), total gene diversity (*H*_T_), interpopulation differentiation (*G*_ST_), and number of substitution types (*N*_ST_).

	*H*_S_	*H*_T_	*G*_ST_	*N*_ST_
Overall populations	0.192	0.878	0.781	0.929*
EH group	0.075	0.714	0.895	0.983*
HM group	0.550	0.922	0.404	0.253
NHM group	0.071	0.498	0.858	0.966*

**Note:**

* Indicates that *N*_ST_ is significantly different from *G*_ST_ (*p* < 0.05).

**Table 4:** Analysis of molecular variance (AMOVA) of chlorotypes for populations and population groups of *T. himalayanum*.

Regions	Source variation	d.f.	SS	VC	Variation (%)	*F*_ST_
Whole distribution	Among populations	19	695.940	3.089	89.43	
	Within populations	218	79.585	0.365	10.57	
	Total	237	775.525	3.453		0.894*
EH group	Among populations	6	186.249	3.253	97.74	
	Within populations	63	4.737	0.075	2.26	
	Total	69	190.986	3.328		0.977*
HM group	Among populations	4	27.756	0.470	39.66	
	Within populations	64	45.794	0.716	60.34	
	Total	68	73.551	1.186		0.397*
NHM group	Among populations	7	358.340	4.269	93.04	
	Within populations	91	29.054	0.319	3.96	
	Total	98	387.394	4.588		0.930*
EH group, HM group versus NHM group	Among groups	1	59.032	0.446	16.20	
NHM group	Among populations within groups	10	214.005	1.907	69.35	
	Within populations	127	50.531	0.398	14.46	
	Total	138	323.568	2.752		0.855*

**Notes:**

* *p* < 0.001.

d.f., degree of freedom; SS, sum of squares; VC, variance component.

### Phylogenetic relationship

**Figure 2:**
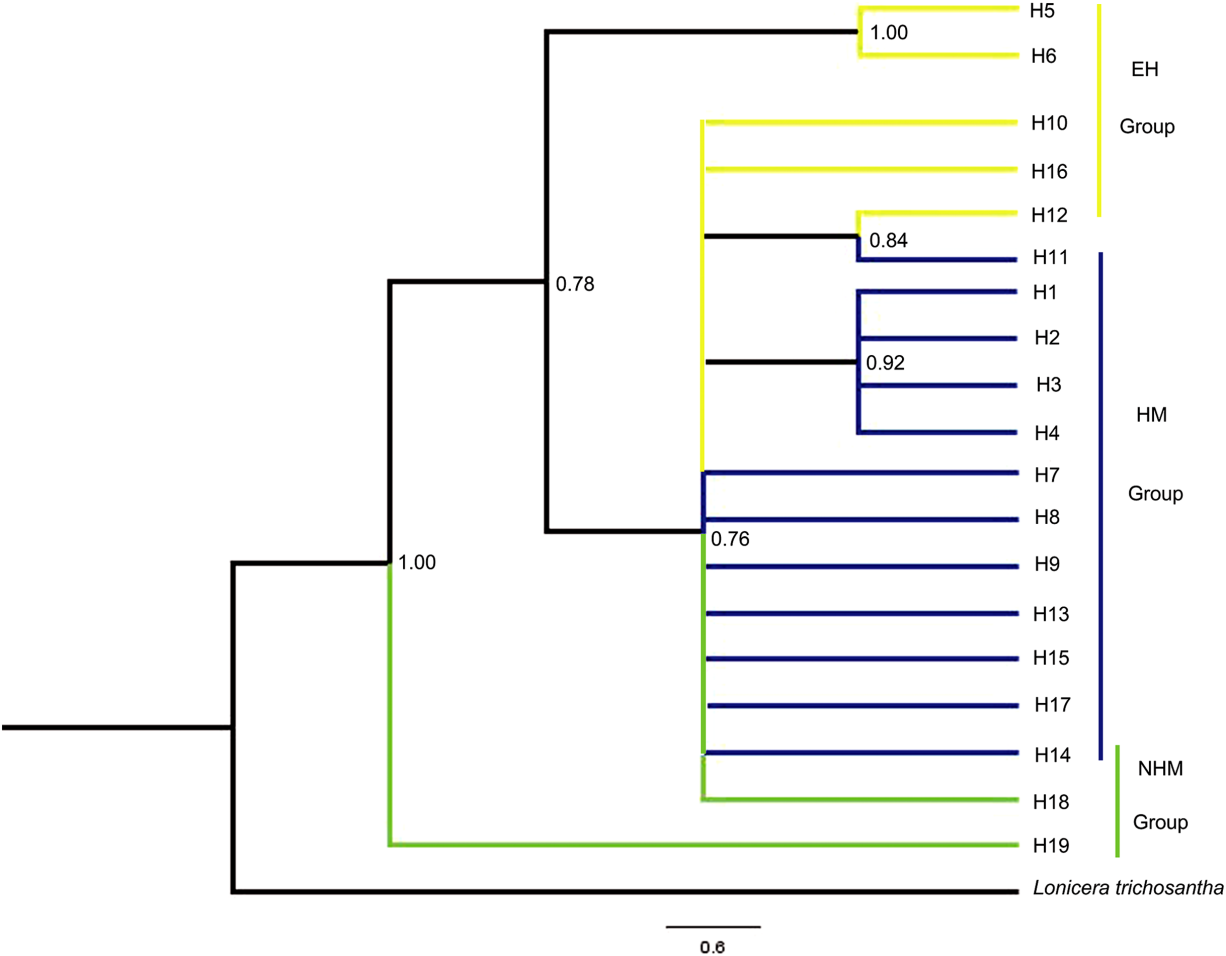
Phylogenetic relationships inferred among the cpDNA haplotypes of *T. himalayanum* based on the sequences of the combined cpDNA from Bayesian inference. Posterior probabilities are given at the nodes.

**Figure 3:**
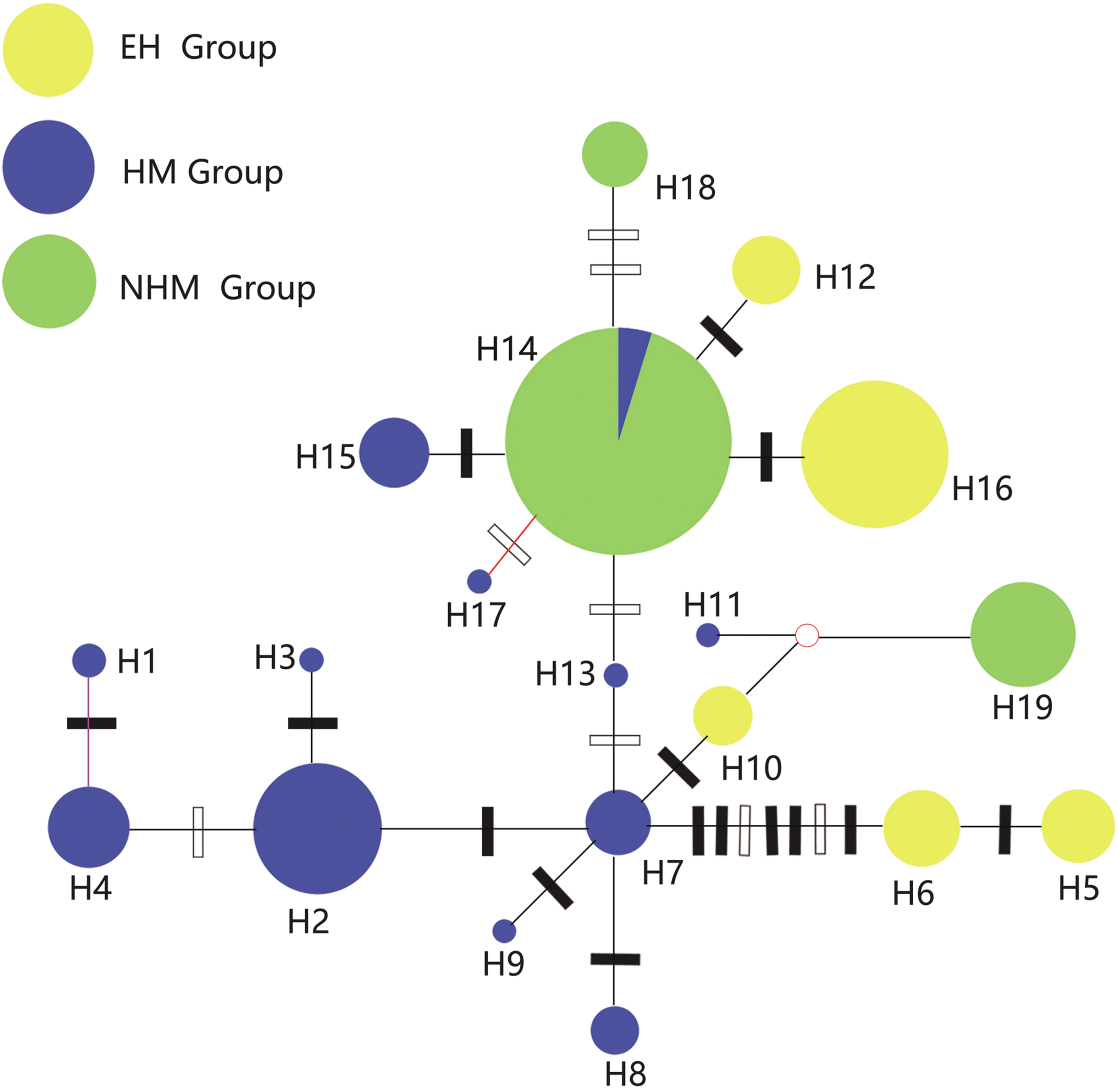
Network of 19 cpDNA haplotypes of *T. himalayanum* (identified by numbers: H1–H19). The size of circles corresponds to the frequency of each haplotype. The small open circles indicate inferred intermediate haplotypes not detected in this investigation. Solid and open bars represent nucleotide substitutions and indels, respectively.

The relationships of the 19 haplotypes are shown in Figs. 2 and 3. Phylogenetic trees constructed using MP, ML, and Bayesian methods are not consistent with each other. They do not show clear relationships between cpDNA haplotypes. However, H19 was observed to occupy the radical position in all trees, and H5 and H6 clustered as one clade in the basal position but only in the Bayesian inference and ML trees. The brush structure observed in the Bayesian tree may be due to the rapid diversification of this species. In the network phylogenetic analysis, the number of mutations between H6 and H7 was seven, illustrating considerable differentiation between the EH group and HM group.

### Demographic history

**Figure 4:**
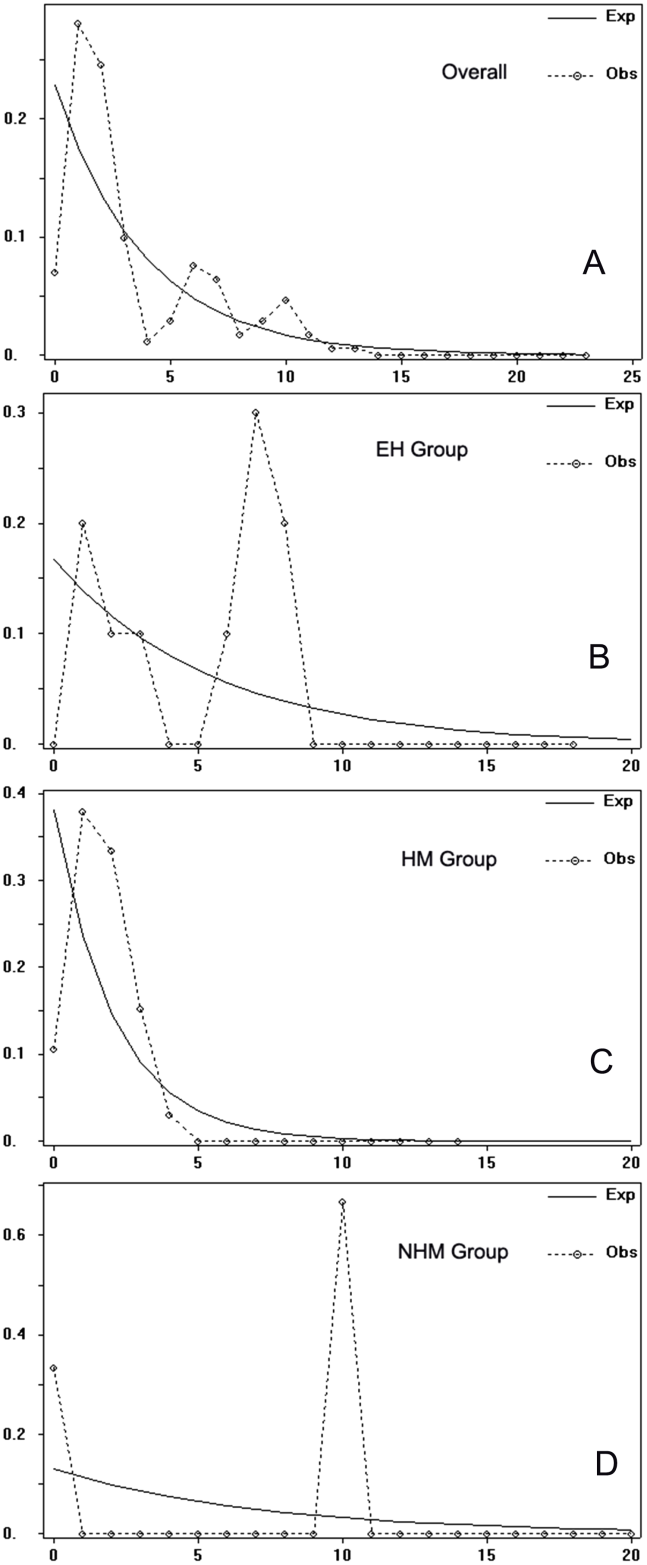
Mismatch distribution in the overall populations and the three groups for chloroplast DNA sequence data of *T. himalayanum*. (A) Mismatch distribution for overall populations. (B) Mismatch distribution for EH group. (C) Mismatch distribution for HM group. (D) Mismatch distribution for NHM group. Solid lines represent expected differences among sequences, whereas dashed lines are drawn from the observed differences.

Tajima’s *D* values of the overall populations and the HM group were negative but not significant (*p* > 0.05), suggesting slight range expansion. Fu’s *Fs* statistics showed positive values or not significant negative values for the whole gene pool and three groups (Table 5). Mismatch distributions of the overall populations, the EH group and the NHM group were multimodal, indicating a demographic equilibrium; the HM was unimodal (Fig. 4). A star-like pattern network is considered to indicate a demographic expansion (Hudson, 1990), thus supporting the findings of the Tajima’s *D*. H7 and H14 are more likely to be the ancestral haplotypes because they occupy a relative central position in the network (Carbone & Kohn, 2001).

**Table 5:** Results of neutrality tests and mismatch distribution analysis for the overall populations and three regional groups of *T. himalayanum* based on the cpDNA dataset.

	Tajima’s *D* test		Fu’s *Fs* test	
Populations	*D*	*p* value	*Fs*	*p* value
Overall	−0.60499	0.30700	2.42674	0.78800
EH group	2.34795	0.99000	9.98669	0.98600
HM group	−0.60620	0.31700	−2.49952	0.16000
NHM group	0.28687	0.66000	22.46008	0.99800

### Paleaodistributional reconstruction

**Figure 5:**
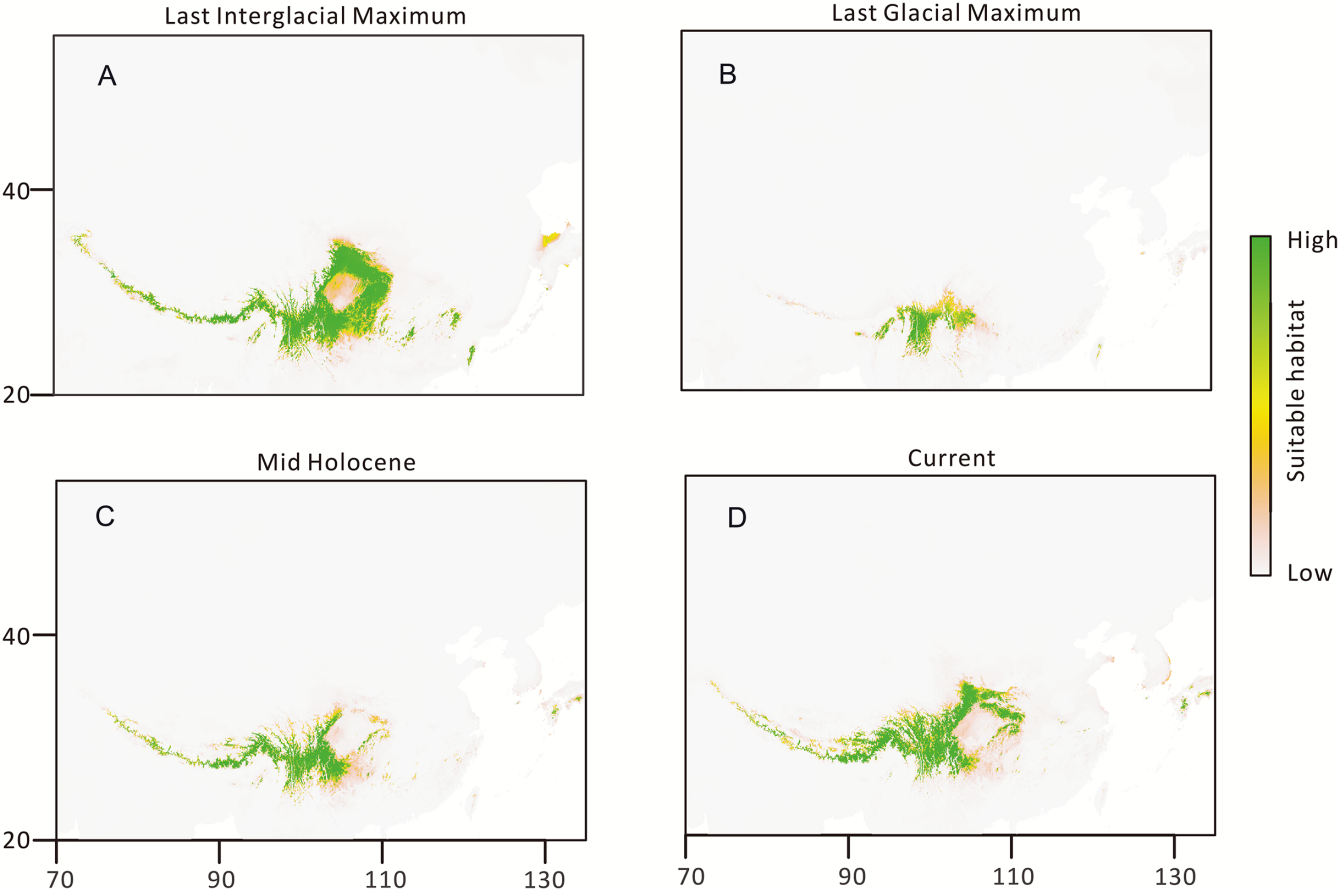
Estimated climatic niche models for *T. himalayanum* distribute at QTP. Maps are shown for each of the four periods tested. (A) Suitable areas for LIG. (B) Suitable areas for LGM. (C) Suitable areas for Mid-Holocene. (D) Suitable areas for Current.

The Palaeodistributional reconstruction showed that during the last interglacial maximum *T. himalayanum* occupied large scope of Himalaya, almost the whole of Hengduan Mountains area, and adjoining region arounding the Sichuan Basin (Fig. 5). During the last glacial maximum, the species was restricted southwards with sharp reduction of distribution, and there were few distribution in East Himalaya and North Hengduan Mountains. From Mid Holocene up to the current the species is expanding Northwards and Westwards again. Climatic niche during three different times suggesting that this alpine herb went through repeated retreat and expansion, and affected by the severe climatic conditions of the QTP.

## Discussion

Paleoorogeny has dramatically changed the topography and geology of the HHM, leading to complex land conditions, such as high mountains and plains incised deeply by numerous valleys and rivers. Thus, this region presents drastic altitudinal variations ranging from 1,000 m a.s.l. (deep valley floors) to numerous peaks above 6,000 m a.s.l., and it is one of the most ecologically diverse areas in the QTP region (López-Pujol et al., 2011). Furthermore, this region is also influenced by both the Indian and East Asian summer monsoons (Zhang et al., 2012a). During the Quaternary, climate changes, especially glacial-interglacial cycles, greatly affected the HHM environment (Zheng, Xu & Shen, 2002). Topographic diversity and climatic circumstances provide heterogeneous habitats for the biota of this region. Several biomes are present (e.g., alpine tundra, meadow, montane forests, and rainforests), and different biomes can be found on the same mountain simultaneously (Ni & Herzschuh, 2011; Zheng & Chen, 1981). To adapt to environmental and climate modifications, organisms are forced to change their habitats and life cycles or to develop new physical traits (Berteaux et al., 2010). Therefore, species differentiation and dispersal are common in the HHM area (Xu, Li & Sun, 2014; Xing & Ree, 2017). Differentiation usually appears between isolated populations, and dispersal usually occurs between regions with broad physiographic similarities (Gao et al., 2015; Xing & Ree, 2017).

### Phylogeographic structure of *T. himalayanum* and its correlation with heterogeneous habitats and Quaternary climatic oscillation

One characteristic of the geographic genetic structure of *T. himalayanum* is a high level of private haplotypes. Twelve out of 19 detected cpDNA haplotypes were private. High total diversity versus a relatively low within-population diversity is another feature of *T. himalayanum*, and this type of geographic genetic structure is found in many studied alpine species located in the HHM and adjoining regions (Yang et al., 2008; Wang et al., 2009; Zhang, Meng & Rao, 2014; Gao et al., 2016; Wan et al., 2016). The comparisons between the values of *H*_T_ and those of *H*_S_ in the overall populations and the three regional groups suggest high genetic diversity between populations. The AMOVA results also showed high levels of genetic differentiation between populations. These findings indicate a clear phylogeographic structure (Avise, 2004) except for the HM group (with an among-population variation of 39.66%). Factors promoting the among-population differentiation of *T. himalayanum* are likely to be complicated. Among those, fragmented distribution and population isolation caused by historical orogenesis and climatic oscillations could be the main factors. In heterogeneous habitats, diverse elevations, precipitation, temperatures, edaphic factors, wind systems, and ecological selection resulting from competitors, predators, parasites, and perhaps mutualists that might restrict other species’ ranges, could accelerate species differentiation (Renner, 2016; Chozas et al., 2017).

Orogenic activities cause a large geomorphologic adjustment, and hydrologic, and tectonic configurations are produced (Li & Fang, 1999). Studies on palaeoaltimetry of the QTP show a progressive uplift from south to north which is likely to have started during or shortly after the beginning of the collision (about 45–40 Ma; Li & Fang, 1999; Mulch & Chamberlain, 2006). Since then, the QTP has experienced shortening on its north–south axis accompanied by the formation of rough surface features and clockwise rotation in its eastern part (Favre et al., 2015). These result in the formation of north–south oriented valleys/rivers in the southeast plateau (including the Yangtze River, the Langcang River, and the Nujiang valleys), as well as west–east oriented valleys/rivers in the south (e.g., the Yarlung Zangbo River) surrounded by high peaks (Favre et al., 2015), which provide pathways for south–north and east–west migration (Wu, 1979; Yu et al., 2017). Due to its immense size and altitude, the QTP and adjacent highland is closely linked to development of the Asian monsoon system and consequently contributed to climatic changes in this region (Ruddiman & Kutzbach, 1991; Liu & Yin, 2002; Tang et al., 2013). During the glacial-interglacial cycle, many species experienced divergent or “contact” evolution during glacial contractions and postglacial expansions (Avise, 2000; Yu et al., 2015). Based on our survey, the distribution of diverse haplotypes in the HM group may have resulted from several contraction/expansion cycles following the repeated advance/retreat of glaciations. The contraction/expansion process may also result in the accumulation of rich haplotypes within the population. This region may play a diversification role and may even be the origin center of *T. himalayanum*. Successive founder events during postglacial recolonization would have led to reduced genomic variability and areas of genetic homogeneity along the recolonization route (Hewitt, 2004). This phenomenon has been frequently observed in the HHM biota (Opgenoorth et al., 2010; Gao et al., 2016; Yu et al., 2017, and references therein). Thus, the decline of gene diversity from the HM group (*H*_T_ = 0.922) to the NHM group (*H*_T_ = 0.498) could result from the south–north direction postglacial recolonization. This hypothesis was also best supported by the palaeodistributional reconstruction (Fig. 5). Furthermore, complicated topography produced by tectonic movement and Quaternary glacial-interglacial cycles could result in range fragmentation of *T. himalayanum*, leading to reduced gene flow and the initiation of intra-species allopatric divergence (Coyne, 1992; Rice & Hostert, 1993; Hewitt, 2000, 2004). This may explain that eight of the 12 haplotypes in the HM group and four of the five haplotypes in the EH group are private. Most studies on the alpine flora of the HHM and adjacent regions have highlighted the effect of the Quaternary period on intraspecific diversification (Zhang, Volis & Sun, 2010; Gao et al., 2012b; Zhang et al., 2012b; Wang et al., 2013; Li et al., 2013; Khan et al., 2015). Therefore, the high genetic variations among populations of *T. himalayanum* could result from strong bottlenecks and founder effects/genetic drift to fix different alleles in isolated populations of fragmented regions (Birky & William, 2001).

### Demographic history of triosteum himalayanum in the HHM area

Even the values of Fu’s *Fs* and mismatch distributions for the overall populations (Table 5) reject the model of current extensive expansion, the star-like network together with the negative values of Tajima’s *D* for the overall populations indicate a demographic expansion. Additionally, the palaeodistributional reconstruction revealed a clear pattern of recolonization from the Mid Holocene up to current. Therefore, we prefer the indication that *T. himalayanum* did experience range expansion from the core region of Hengduan Mountains (HM group) to both the East Himalaya area (EH group) and the North Hengduan Mountains (NHM group) from Mid Holocene.

The phylogenic relationship of the star‐like network indicates that haplotypes of the EH group originated from Hengduan Mountains areas, and there was also a decline of gene diversity from the HM group (*H*_T_ = 0.922) to the EH group (*H*_T_ = 0.714). One of the migration corridors may be the Yarlung Zangbo Valley, which is the largest vapor channel of the QTP (Lin & Wu, 1990). Lower elevation and intense airflow create ideal circumstances for the dispersal of seed and pollen of *T. himalayanum.* The other important dispersal corridors could be the long, narrow mountain areas. Since the Himalayas are linked to the Hengduan Mountains, exchanges along the mountain ridges or slopes occur easily. This hypothesis has been assumed in many studies and reviews (Cun & Wang, 2010; Luebert & Weigend, 2014; Yu et al., 2015, 2017). As discussed above, the south–north direction of the Hengduan Mountains and the river valley provide channels for glacial contractions and postglacial expansions for plant and animals and facilitate communication between different ecoregions of the HM and NHM areas. Therefore, haplotype H14 is not only shared by both the HM group and the NHM group, but it is also the most dominant and widely distributed haplotype.

However, anthropogenic activities that reduce the distribution range and genetic diversity could be other factors responsible for lower gene diversity and within-population variation of the NHM group in addition to bottleneck or founder effects. Many previous habitats of *T. himalayanum* recorded in documents are now surrounded by folk houses, and we were not able to locate them during the field work conducted two years ago. We should pay more attention to this phenomenon and more effectively preserve this alpine herb for future study and utilization.

## Conclusion

The hypothesis that HHM is both the distribution and diversification center for alpine plants was supported by molecular diversity of *T. himalayanum*. The south–north and east–west migration from core area of HM was inferred from the phylogeographic structure, including the decline of gene diversity from the HM group to both the NHM group and EH group, phylogenetic relationship, and feasible topography and environment for dispersal. The palaeodistributional reconstruction result also showed an extremely clear pattern of retreat and recolonization during last episodes of interglacial and glacial periods.

However, more integrative multidimensional analyses of morphology, geography, ecology, and phylogeny should shed light on this fascinating research region, since the understanding of interactions between varying topography, environment, and the evolution of biota during the Quaternary are far from sufficient.

## Supplemental Information

10.7717/peerj.4748/supp-1 Supplemental Information 1 19 haplotypes sequences. Haplotypes (H1–19) detected among 20 populations of *Triosteum himalayanum.*.

## Additional Information and Declarations

### Data Availability

The following information was supplied regarding data availability: GenBank: MH122760–MH122783.
